# Ultrasound in GCA: Halo Sign Quantification and Visual Symptoms, Systemic Inflammation and Relapse Risk

**DOI:** 10.31138/mjr.080823.hsv

**Published:** 2024-01-25

**Authors:** Gen Nen Ho, Laura Devonshire, Rainer Klocke

**Affiliations:** 1 Department of Rheumatology, Dudley Group NHS Foundation Trust, United Kingdom; 2 Department of Vascular Sonography, Dudley Group NHS Foundation Trust, United Kingdom

**Keywords:** giant cell arteritis, temporal artery ultrasound, ocular ischaemia

## Abstract

**Background:**

A sonographic scoring system, termed Halo count and Halo score, of temporal and axillary arteries (TAXA) in suspected giant cell arteritis (GCA) has been proposed for outcome prognostication.

**Method:**

We conducted a retrospective review into the relationship of Halo count and Halo score and clinical-laboratory parameters amongst patients diagnosed with GCA via our rapid-access pathway to determine whether these measures should form part of our local routine clinical practice.

**Result:**

This review of TAXA ultrasound (US) images in patients with diagnosed GCA did not identify any correlation between Halo count/score and ocular symptoms, jaw claudication, 6-month relapse risk or inflammatory markers.

**Conclusion:**

This suggests that further prospective evaluation of Halo count and -score is required before adopting these measures into routine US scanning of TAXA for suspected GCA.

## INTRODUCTION

The diagnosis of GCA has been traditionally confirmed via temporal artery biopsy (TAB). However recent years has seen an increased use of TAXA US and its incorporation into major guidelines (BSR,^1^ ACR/EULAR2) in diagnosing GCA as literature suggests a comparable sensitivity and specificity with TAB.^3^ TAXA US is also less invasive, more readily available, and faster to perform than TAB in most healthcare settings. The sonographic hallmark of GCA is defined as a non-compressible halo (concentric, hypo-echoic area around the vessel lumen) representing a thickened inflamed intima-media complex.^4^

A sonographic scoring system known as Halo count and Halo score has been proposed by Van der Geest et al.^5^ for prognostication of GCA: a Halo count ≥2 and Halo score ≥3 were shown to be associated with 30% risk of ocular ischaemia compared with ≤ 5% for lesser count/scores.

Our objective was to investigate the relationship of Halo count and Halo score and clinical-laboratory parameters amongst patients diagnosed with GCA via our local rapid-access pathway to determine whether these measures should form part of routine clinical practice at this Trust.

## MATERIALS AND METHODS

In this retrospective study, 31 consecutive patients with a clinical diagnosis of GCA and supportive TAXA US scans (using a Philips Epiq 5G US system with a linear probe: L18-5 MHz range, 158 Hz wall filter) between February 2019 and September 2021 at Dudley Group NHS Foundation Trust were identified for inclusion in this study.

All authors participated in the evaluation of TAXA US still images (LD and RK as experienced sonographers; GNH as a novice). For the first 13 patients, we jointly reviewed the images in the database to standardise our methods in defining the Halo sign (as per Schmidt^4^) and measuring Halo thickness, and grading according to a Grade 0–4, suggested by Van der Geest et al.^5^ Halo count (ie, number of arteries with Halo sign, maximum of 8, namely common temporal, frontal and parietal temporal artery branch, and axillary artery on both sides) and Halo score (total Halo grades of all 8 artery sections, with a maximum score of 48, due to axillary arteries being given higher weightage) were then calculated based on Van der Geest’s formula. For the second 19 patients, the three investigators measured Halo thickness and graded individual arteries independently. Interobserver agreement between two experienced sonographers was 74.1% for perfect agreement and 100% for agreement within 1 Halo grade difference. Halo grade differences between observers were resolved by using the majority (where at least 2 observers agree) or the average of the 3 grades for Halo score calculation. Clinic letters were reviewed for patient characteristics, such as inflammatory markers, ischaemic ocular symptoms (IOS, ie any recorded visual symptoms, transient or permanent), and relapse of GCA within 6 months of diagnosis, for correlation with Halo count and score.

Patient characteristics are presented as number of patients and percentage of total sample. Correlation between Halo count/score with IOS, jaw claudication, relapse within 6 months were determined using Mann-Whitney U Test. Spearman Rank correlation were used to demonstrate the spread of Halo count/score versus CRP/ESR values.

## RESULTS

Of 31 identified patients, 29 (94%) had cranial GCA, while 2 (6%) were diagnosed as extra-cranial GCA; 7 (22.6%) had relapse at 6 months. Further patient characteristics are shown in **Table 1**. Median Halo count was 2 (IQR 2,4) and median Halo score 10.5 (IQR 7, 19.5). Sixteen (51.6%) patients had commenced high-dose steroid a median 3 days (IQR: 2 days, 7 days) prior to clinical and US assessment, while one patient was already established on low dose steroid for PMR prior to GCA diagnosis. The proportion of patients with IOS was no higher in the high In 22 (71.0%) patients, the report did not specify whether axillary arteries were scanned. (this group of patients are shown as AxN in the subsequent results).

**Table 1.** Patient characteristics at presentation.

**Patient characteristics**	**Number of patients (n=31)**
Sex, no. of males [%]	10 (32.2%)
Age, median (range)	76 (66–89)
Steroid prior to ultrasound [%]	17 (54.8%)
Temporal headache [%]	16 (51.6%)
Scalp tenderness [%]	17 (54.8%)
Jaw claudication [%]	15 (48.3%)
Visual symptoms [%]	13 (41.9%)
PMR [%]	8 (25.8%)
Constitutional symptoms [%]	14 (45.2%)
Ischaemic ocular symptoms (IOS) [%]	6 (19.3%)
ESR, mm/hour, median (range)	82 (22–147)
CRP, mg/L, median (range)	71.5 (3–349)

**Figure 1.**
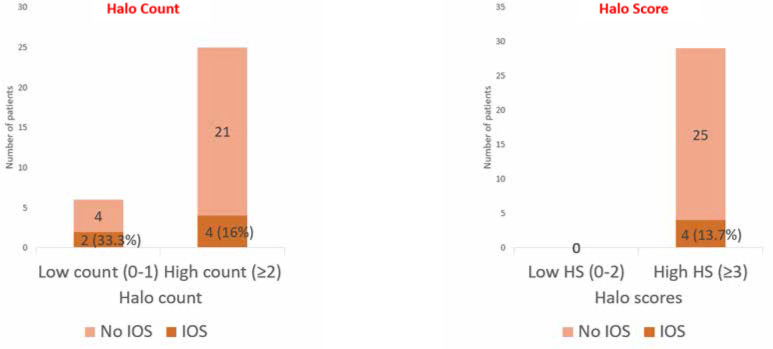
Proportion of subjects with ischaemic ocular symptoms (IOS). IOS in low vs high Halo count group **(left)**, IOS in low vs high Halo score group **(right)**.There was no significant difference in Halo count or score according to jaw claudication or risk of relapse at 6 months either (see **Figure 2**).

**Figure 2.**
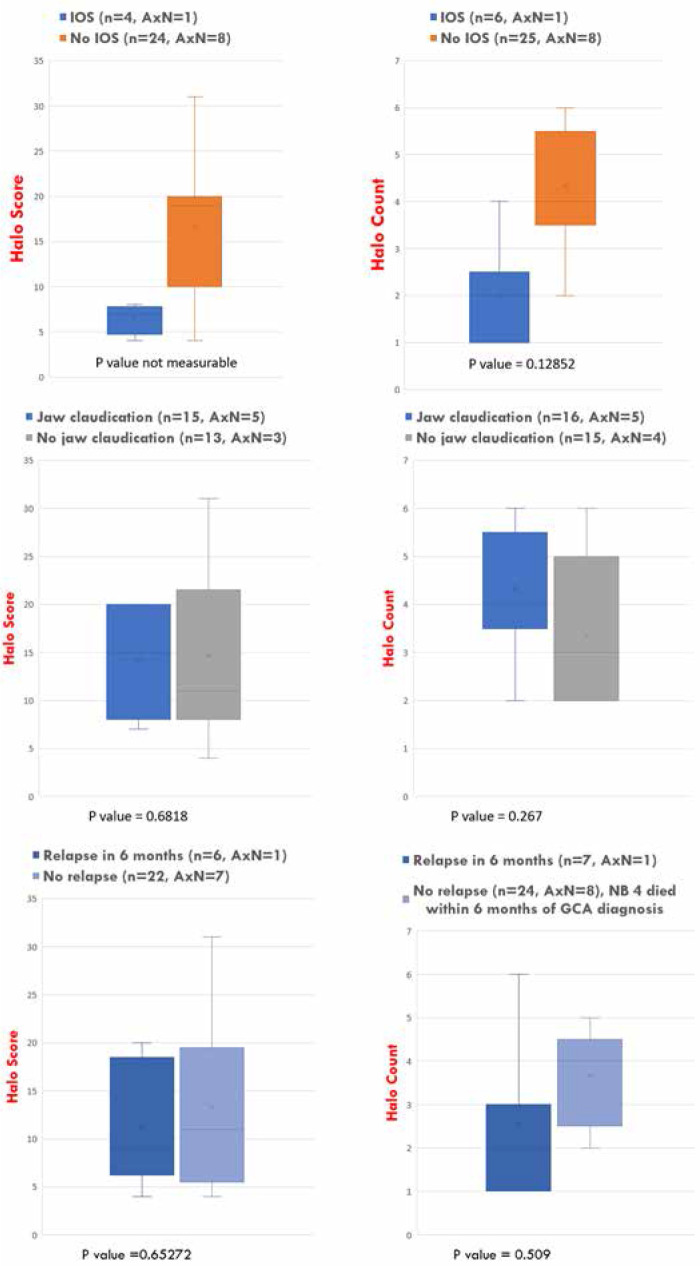
Halo score/count according to ischaemic ocular symptoms (IOS), jaw claudication, and relapse of GCA within 6 months. P-values are based on Mann Whitney U testing between groups. No correlation was found between Halo count/score and acute phase response (CRP and ESR) (see **Figure 3** and **Figure 4**).

Halo count group **(Figure 1)**. Median Halo count in patients with IOS (n=6) versus those without IOS were 2 (IQR 1, 2.5) and 3 (IQR 2, 4.5), respectively **(Figure 2)**.

**Figure 3.**
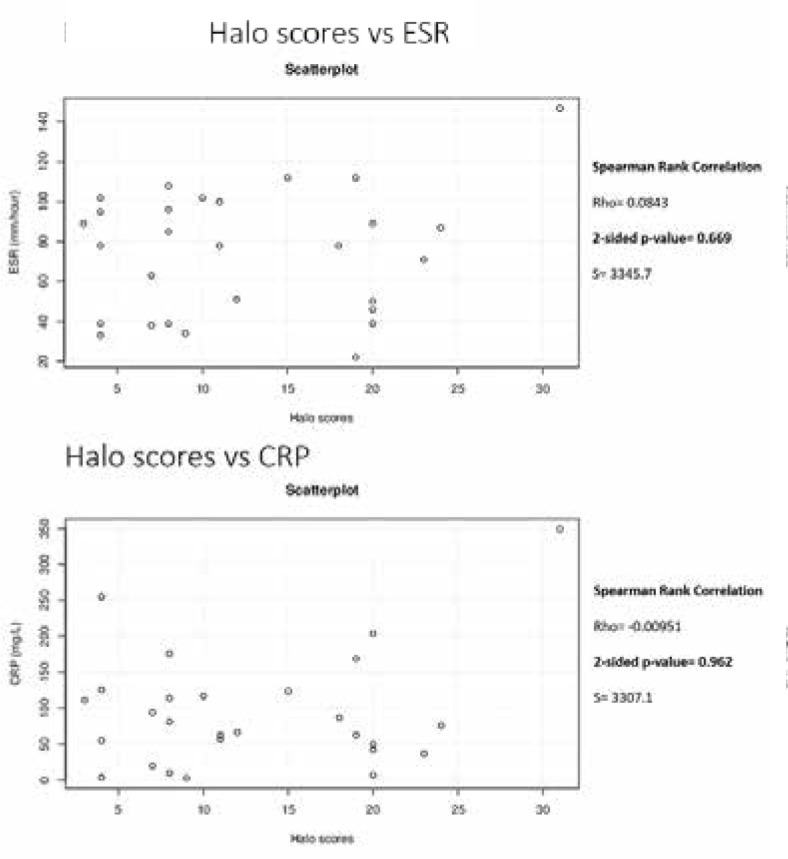
Halo scores vs ESR and vs CRP.

**Figure 4.**
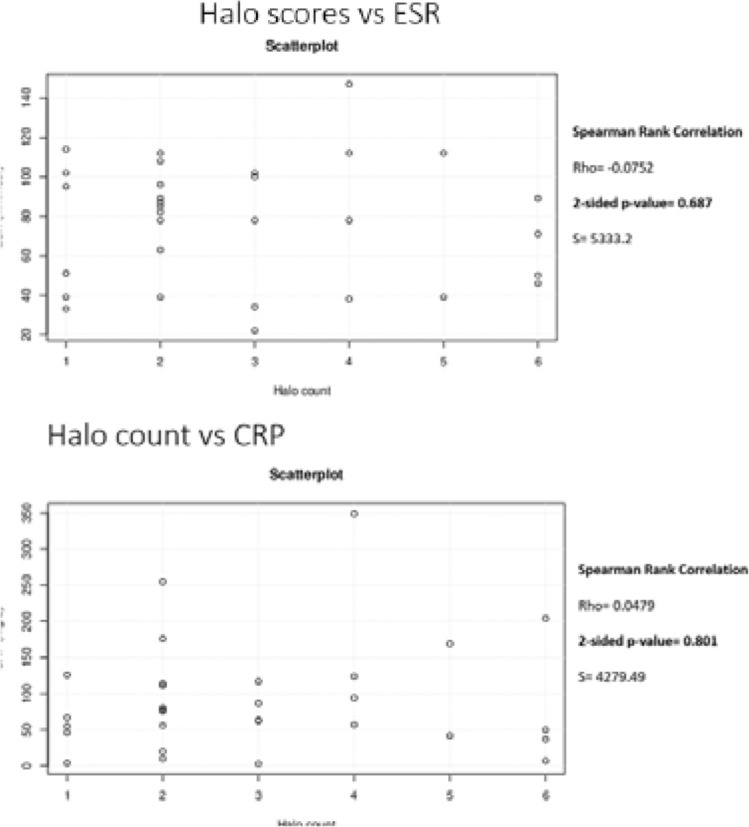
Halo counts vs ESR and vs CRP.

## DISCUSSION

Van der Geest et al.^5^ demonstrated in patients with suspected GCA that the extent of vascular inflammation on ultrasound, that is, a Halo count ≥2 or Halo Score ≥3, identifies GCA patients at higher risk for ocular ischaemia: >30% vs ≤5% with lower Halo count/scores. In addition, they found a positive correlation of Halo counts and Halo Scores with systemic inflammation, ie ESR and CRP, a finding subsequently confirmed also by Collada et al.^6^ Our retrospective evaluation of TAXA US images in patients with diagnosed GCA did not identify any correlation between Halo count or score and ischaemic ocular symptoms, jaw claudication, 6-month relapse risk or inflammatory markers. If anything, patients with ischaemic ocular symptoms had lower Halo scores and count.

There were key methodological differences between our review and the previous prospective studies: In Van der Geest et al.^5^ US data included patients with suspected GCA and GCA diagnosis was determined at 6 months, taking into account the initial TAXA US findings and consecutive clinical assessment. In our study, patients were selected based on a supportive US scan alongside a clinical diagnosis of GCA at presentation. Furthermore, Van der Geest et al.^5^ prospectively scanned temporal and axillary arteries in a systematic fashion to form the basis of the proposed halo count and halo score, whereas we applied these measures retrospectively on the available recorded images of TAXAs. These images included the Halo-positive TAXA vessels underpinning the US conclusions but not all arteries examined. In 29% of patients, scanning of the axillary artery was not specifically mentioned or recorded as images. This is a significant difference and limitation of our study. Our data sample size is also smaller compared with the Van der Geest^5^ study which could explain the lack of correlations with clinical and laboratory markers seen. 51.6% of patients in our cohort were commenced on high dose steroid prior to TAXA US which may have an impact on the sonographic finding and inflammatory markers.

Acknowledging these limitations of our data, our retrospective study has provided useful local information. We would suggest that further prospective evaluation of Halo count and score is recommended before adopting the more time-intensive, systematic Halo score/count measurements into routine clinical temporal and axillary US scanning for suspected GCA.
